# Challenges Associated with Informed Consent in Low- and Low-Middle-Income Countries

**DOI:** 10.3389/fvets.2016.00092

**Published:** 2016-10-20

**Authors:** Melissa Upjohn, Kimberly Wells

**Affiliations:** ^1^Brooke, London, UK

**Keywords:** informed consent, LMICs, NGO, equine, welfare, animals

## Abstract

Obtaining informed consent from research participants is a generally recognized step of undertaking research. While the concept of informed consent is well understood in western research environments, it requires further consideration when reviewing studies involving humans and owned animals in low- and low-middle-income countries (LMICs), in order to take account of different social, educational, and research norms. Here, we identify some of the challenges that need to be considered, and how they might affect the process of obtaining informed consent. We explain the approach taken by an animal welfare non-governmental organization working in LMICs to addressing these challenges. There are also questions that reviewers might consider when commenting on work originating in this context.

## Introduction

Frontiers in Veterinary Science author guidelines endorse the Helsinki declaration and guidelines of the International Committee of Medical Journal Editors. These include the requirement to obtain informed consent from all research participants. In this perspective piece, we reflect on challenges encountered in achieving informed consent for research undertaken by an international non-governmental organization (NGO) working with people and animals in low- and low-middle-income countries (LMICs) as defined by the World Bank (1, 2). We describe the context in which the NGO works before outlining the research activities that complement programmatic and advocacy activities. After identifying consent-related challenges, we then consider how potential difficulties faced in demonstrating adherence to the Helsinki declaration are addressed by this NGO. We also propose how those who review manuscripts describing work originating from LMIC contexts can take account of local limitations in their feedback.

## The Research Context

There are 113 million horses, donkeys, and mules, collectively called equids (3), worldwide. Approximately 100 million of these are in LMICs where they are working animals undertaking a wide range of commercial and domestic tasks (4). Few peer-reviewed studies of their contribution to human livelihoods have been published, but field research indicates that they fulfill an essential role in their owners’ and users’ daily lives. They undertake income generating activities that enable families to buy food, essential non-food items, health care, and education services. By facilitating tasks, such as sourcing water, animal forage, and firewood, they reduce the physical burden involved, primarily for women, and free up time for childcare (5). In contrast with the well-resourced context of most sporting and pleasure horses, many working equids are found in very low resource settings. Lack of access to appropriate food and water, poor or inaccessible health care, lack of owner understanding and funding to meet essential equine needs, combined with the often extreme conditions in which they and their owners work, result in these equids suffering a range of adverse welfare issues, including wounds, heat stress, malnutrition, and poor handling (6). The inter-related nature of these resource constraints make identifying the root causes of welfare problems challenging. The identification of local, sustainable solutions is generally more complex than simply advising owners, for example, to work less, give more water or feed better quality and greater quantities of food.

Brooke is an equine welfare NGO that works in Asia, Africa, and Latin America to address welfare issues affecting these animals. It operates directly or through partner organizations and local service providers to ensure the availability of equine-specific health services and ancillary services, such as farriery and saddlery; it undertakes community engagement to build understanding among owners and users of equine welfare needs, to develop appropriate handling and husbandry practices and to facilitate social change in building momentum toward wider community recognition of equids’ needs as sentient animals. Through advocacy work, it engages with policy makers to promote recognition of the value that equids contribute to household and community livelihoods and to ensure the inclusion of equids in local, regional, and national animal services planning and provision.

## NGO Research Activities

To underpin its programmatic and advocacy-related work, Brooke undertakes two forms of research.

Animal-based research involving non-invasive examination of owned equids, equipment evaluation, or observation of human–animal interaction. Research is designed to develop a better understanding of causal factors associated with a welfare problem, clinical issues facing service providers or to test a novel intervention in a field setting to investigate if it achieves the desired welfare benefit. Examples of such research that have been shared *via* peer-reviewed publication and/or scientific conference presentation can be viewed on Brooke’s website: https://www.thebrooke.org/for-professionals/research-publications-and-conference-presentations.

Human-based research involving either a one to one interview or a focus group discussion. This aims to understand attitudes and/or practices of owners, users, or service providers or to define the contribution derived from equids to human livelihoods and daily lives. Alternatively, it may involve investigating the relationship between animal and human welfare.

All of Brooke’s research involves interacting with equids and/or people in the field and is designed to build Brooke’s evidence base for its activities. Since most is “action research,” (7) whose results are designed to be applied within programmatic activities, the animals and people involved in research are also the beneficiaries of the services offered and the people with whom community engagement activities are undertaken. This point is also referred to by the Wellcome Trust research guidance notes for involving people in LMICs (1, 2).

Both service provision and community engagement activities are developed on the basis of a long-term relationship of mutual trust with owners, users, and other local people. Identification of priority research topics by the community and staff working with them and the incorporation of results into programmatic activities rely on the local community’s ownership of the research and its relevance to their lives. If data need to be collected before this relationship has been allowed sufficient time to develop, it is possible that data will be of lower quality due to lack of understanding by researchers of the local context, and by the community of the value of the research for their livelihoods.

## Challenges to Achieving Informed Consent in This Context

Working through the key aspects of informed consent identified in the World Medical Association guidance (8), a number of questions arise:

“Individuals must be capable of giving informed consent.”

How is “capable” defined? Two key aspects of capacity must be addressed, namely legal capacity and cognitive capacity. Does the person from whom you are seeking consent own the animal and how is ownership evidenced? If (s)he does not own the animal, how is the owner’s consent sought? What impact does any lack of local legal framework, which defines responsibility for an animal’s welfare, have on an owner’s willingness to agree to participate in research? How does the person’s educational background impact their ability to understand what is being explained? What level of literacy does (s)he have and how does this affect the way in which information about the research is communicated? As animals are property and the animals themselves cannot give informed consent, owners are required to make good judgments about their animal’s involvement. How realistic is this in a situation where recognition of animal welfare may be less widespread and owners’ appreciation of animal behavior may be limited? When we undertake animal examination during routine field work to assess welfare, if the animal behaves in such a way that a particular measure cannot be recorded, does this imply “refusal of consent” by the animal?

“Participation must be voluntary, agreement must be freely given.”

How can this be ensured in a context where there is an existing dependency? As noted earlier, action research is by definition conducted in the community within which relationships are ongoing. What effect does asking people to participate in research have on existing relationships with them and their fellow community members? Do they really feel empowered to say “no” to the request? What is their understanding of whether their reply will affect their ability to access services in future? How does being asked to participate, regardless of whether they say yes or no, affect their willingness to continue being involved with community engagement activities?

“Each subject must be informed of key information: aims, methods, sources of funding, conflicts of interest, institutional affiliations of researcher, potential benefits and risks to participants, potential discomfort, post study provision, other relevant information.”

How is this information transmitted effectively to participants who may be poorly educated and/or illiterate? How is understanding of the research verified? How can the breadth of consent be recorded if a participant cannot sign their name? Many of the individuals with whom this research is undertaken have to live from 1 day to the next and may not be used to considering longer term consequences of current activities. These cover both the longer term application of the data being collected and its dissemination *via* electronic media for both scientific and, in the case of an NGO, communications and fund raising purposes. Some communities have mobile phones and access to social media so they are increasingly able to access outlets where summary narrative or pictorial research outputs may be published. It is essential that they are comfortable with research in which they participate being potentially shared in this way.

“Each subject must understand their right to refuse to participate or to withdraw agreement at any time without reprisal.”

How can this understanding be ensured and maintained in the context of existing dependency? How do we address the resultant withdrawal bias if owners and/or their animals decline to participate?

“After ensuring understanding, seek consent, preferably in writing. If written consent isn’t possible, witness of non-written consent must be formally documented and witnessed.”

If participants are illiterate how can this be achieved and what approach is ethically correct for us to adopt if their normal way of doing things is different from this? In many countries in which working equine NGOs operate, activities that appear “official” in terms of gathering data or asking a person to sign a piece of paper may be viewed with suspicion or even outright hostility. For example, government data collection can be associated with future tax liability assessment. People may view signing anything that looks “official” as potentially committing them to something other than answering questions or agreeing to have their animal examined. This is particularly difficult to address in situations where literacy limitations mean that it is not possible for the person to read the document they are being offered as explanation of the research or their agreement to its terms. Nor is it possible for them to read an example of the kind of output that may arise from the research, to enable them to appreciate how what seems like a “day to day conversation” that is a normal part of programmatic interactions may be described more formally to others. Outputs, such as a conference abstract, a published paper, or potentially even a description on social media, can vary in tone and content, so these differences are hard to convey verbally. What are owners’ concerns and priorities in respect of how results are shared and how do they voice them? It may also be difficult for participants to understand what the distinction is between a given “conversation” requiring completion of a consent process and many other such “conversations” which do not. What arrangements to witness understanding and consent are feasible in such circumstances?

“All subjects should be given the option of being informed of general outcome and results of study.”

What mechanisms are in place to communicate study findings to individuals and the wider community? How are community members involved in dissemination of the results of a study and how do we balance the sharing of results against using up more of their valuable time for this part of the activity?

“If seeking consent from a participant in a dependent relationship with the researcher, consent must be sought by a qualified individual who is independent of this relationship.”

In an ongoing dependency relationship, how can an independent individual be found who the participant trusts?

“For a subject incapable of giving consent, this must be sought from a legally authorized representative.”

This could apply to both children and animals, as mentioned above. How can legal authority be proved, and what are the local norms regarding who is legally responsible for them and under what circumstances?

## Addressing the Challenges

Informed consent of working equid owners forms part of autonomy in a biomedical ethical framework (9). As noted by Laws et al. (10) who described research in international development, and Whay (11) who noted the specific ethical challenges of research involving working equids, the multiple contextual components of informed consent must be recognized and addressed, see also Wellcome Trust guidance (1, 2).

Rather than approaching them on an item by item basis, Brooke adopts a holistic approach, while recognizing that it must be possible to demonstrate how the key requirements of the Helsinki agreement have been addressed.

Brooke aims to embed a culture of care for the animals and owners with whom we work in all staff. Although Brooke’s research is not anthropological, primary principles described in the guidelines of the Association of Social Anthropologists (12) offer close parallels with our work. They refer to “close and often lengthy association of anthropologists with the people amongst whom they carry out research… trust and reciprocity between the research and research participants; it also entails recognition of power differentials between them.” In seeking to protect people and animals and honoring trust, Brooke has established an Animal Welfare policy that sets out non-negotiable practices relating to research with equids and their owners and users.

These practices ensure that the welfare of the animals and their owners are paramount. All members of staff are charged with ensuring that the risks to animals and people as a result of Brooke’s activities are minimized. The policy states that country program staff is responsible for seeking informed consent and ensuring that the dignity, rights, safety, and well-being of research participants are considered and that any potential risks are mitigated.

All Brooke staff and consultants are required to adhere to the policy. Senior management is charged with ensuring adherence of their staff.

Brooke has an Animal Welfare and Ethical Review Body (AWERB). In addition to a chair and executive secretary role, membership is comprised of five advocate roles that are appropriate to organizational needs while also fulfilling minimum requirements specified by the UK Home Office: a Named Animal Care and Welfare Officer, Named Veterinary Surgeon, Named Training and Competence Officer, scientific and lay members (13). The animal welfare, veterinary, study design, people (i.e., human welfare), and public (i.e., lay) advocates are charged with reviewing research proposals and protocols using people and/or animals as subjects to ensure appropriate compliance with ethical principles. They do so by championing relevance and rigor of the study, public perception of our work and principles included within concepts, such as One Health, Five Domains of Animal Welfare (14), and Helsinki declaration.

The policy states that country program staff must inform all participants of the intended use of the research and ensure they have the opportunity to receive feedback on the results of research in which they are involved. In discussion with the UK technical support team, a country program team planning research develops a protocol, which includes a description of any welfare issues arising, how they will be mitigated and how consent will be sought from all participants. They agree how personal data will be handled for legal data protection purposes, using templates that can be adapted to suit the individual context. AWERB reviews this proposal and comments on any aspects requiring clarification or amendment prior to the research being approved. Once research has been completed, AWERB reviews the outputs and outcomes and includes participant consent and feedback processes in this review. While this process is imperfect, it is hoped that it enables lessons to be learned from each piece of research, which can be incorporated into future research activities.

## Peer Review of Field Studies from LMICs

Peer-reviewed journals establish a rigorous review process for ensuring the quality of the research that they agree to publish. In general, peer reviewers are required to focus on the aspects of study design, the research question asked, data collection, and the analysis of those data in determining quality. For studies in LMICs, there may be particular logistical challenges, which need to be acknowledged when doing this and in what can be reasonably expected. For studies undertaken under the auspices of a university the ethical review process is generally defined, although in LMICs it may follow different principles from those applied by western universities due to cultural differences in recognition of both human and animal welfare. Understanding of informed consent is generally incorporated into a standardized question regarding the ethical review process during the manuscript submission process. However, the challenges described here may mean that, where research is undertaken in LMIC settings, it is more appropriate to include questions about the process of obtaining informed consent within peer review processes. This applies regardless of whether the research is undertaken by a research institution or an NGO.

## Conclusion

The context in which field research in LMICs is undertaken may differ substantially from that generally encountered by many peer reviewers for a journal, such as Frontiers in Veterinary Science. In addition to considering the logistical issues relating to study design for research, recognition of the challenges associated with obtaining, and recording informed consent is required when reviewing such studies for publication.

## Author Contributions

MU originated the concept for the article, prepared first draft of the manuscript, coordinated contributions from second author. KW contributed to the concept for the article and amended the draft manuscript.

## Conflict of Interest Statement

The authors declare that the research was conducted in the absence of any commercial or financial relationships that could be construed as a potential conflict of interest.
